# Relationship between Chemical Structure and Biological Activity Evaluated In Vitro for Six Anthocyanidins Most Commonly Occurring in Edible Plants

**DOI:** 10.3390/molecules28166156

**Published:** 2023-08-21

**Authors:** Izabela Koss-Mikołajczyk, Agnieszka Bartoszek

**Affiliations:** Department of Chemistry, Technology and Biotechnology of Food, Faculty of Chemistry, Gdańsk University of Technology, 80-233 Gdańsk, Poland; agnieszka.bartoszek@pg.edu.pl

**Keywords:** anthocyanidins, anthocyanins, chemoprevention, antioxidant activity, cytotoxicity, genotoxicity

## Abstract

Numerous studies have provided evidence that diets rich in anthocyanins show a broad spectrum of health benefits. Anthocyanins in nature are usually found in the form of glycosides. Their aglycone forms are called anthocyanidins. The chemical structure of anthocyanins is based on the flavylium cation, but they differ in the position and number of substituents. However, the bioactives and foods that contain them are frequently treated as a uniform group of compounds exhibiting the same biological activity, without paying attention to the structural differences between individual anthocyanidins. The aim of this study was to find out how structural differences impact the biological activity of the six most common dietary anthocyanidins, i.e., delphinidin (Dp), petunidin (Pt), cyanidin (Cd), malvidin (Mv), pelargonidin (Pg) and peonidin (Po). The study concentrated on redox-related phenomena and compared the following parameters: antioxidant activity (measured using various methods: spectrophotometric tests (ABTS, DPPH), ORAC assay and CAA test (cellular antioxidant activity)), the ability to inhibit growth of human colon cancer cells (HT29; determined using MTT assay), and the ability of studied compounds to protect DNA from oxidative damage (comet assay). Based on the obtained results, the relationship between the structure of studied anthocyanidins and their biological activity was assessed. The obtained results revealed that the number and position of the hydroxyl and methoxy groups in the anthocyanidin structure strongly influenced not only the color of anthocyanidins but most of all their antioxidant and biological activities.

## 1. Introduction

Anthocyanins are natural pigments found in plants, especially in flowers, tubers, fruits, and leaves [1]. They give plants colors from blue and purple to red [2]. Anthocyanins are characterized by a C6-C3-C6 carbon skeleton. They mainly occur in plants in the form of polyhydroxy and polymethoxy glycosides derived from the flavylium cation. Structural differences between anthocyanins are related to the number of hydroxyl or methoxy groups in the anthocyanidin backbone, the position and number of saccharide residues, and the aliphatic or aromatic acids bound to them [3].

**Figure 1:**
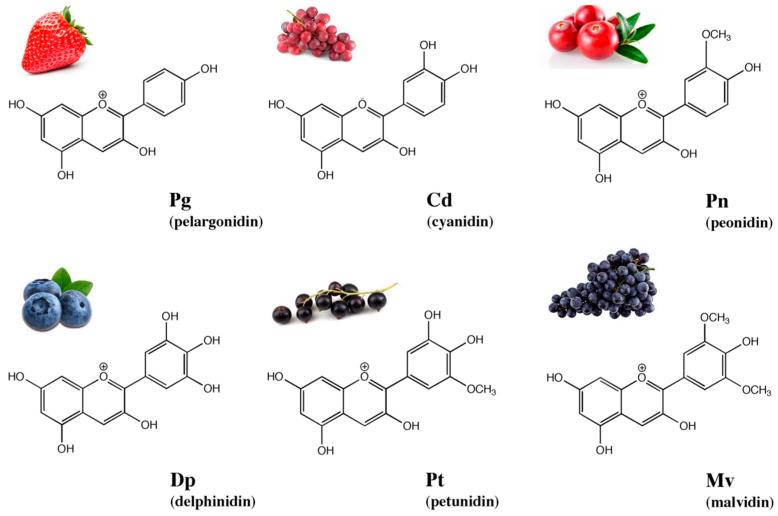
Chemical structures and examples of the natural sources of the six most common anthocyanidins. Source: created by the Authors using BioDraw Ultra 10.0 software (PerkinElmer, Waltham, MA, USA).

The aglycone forms of anthocyanins are called anthocyanidins. To date, over 1000 structurally distinct anthocyanin molecules and approximately 27 structurally distinct anthocyanidins have been identified [4]. However, only six of them account for 90% of anthocyanins found in nature. The six most common anthocyanidins are cyanidin (Cd), malvidin (Mv), pelargonidin (Pg), delphinidin (Dp), petunidin (Pt) and peonidin (Pn) [5]. The differences between their chemical structures result from the different number and position of the methoxy and hydroxyl groups in the structure of the flavylium ion (Figure 1) [5].

Numerous studies, mostly carried out in cell culture models, have shown that anthocyanins exhibit a wide range of health-promoting properties, including anti-inflammatory, antioxidant, antimicrobial, and anticarcinogenic activities [4,5,6]. The latter activity has been determined in vitro against different human cancer cell lines. Based on these studies, anticarcinogenic mechanisms of action of these compounds were proposed, including downregulation of cyclin-dependent kinases, enhanced expression of p21, activation of caspase 3, inhibition of MMP (matrix metalloproteinase) activity, and lessening Snail and pSTAT3 (phosphorylated signal transducer and activator of transcription) expression. Wu et al. [7] investigated the growth-inhibitory properties of delphinidin towards the HER-2-positive MDA-MB-453 breast cancer cell line. They demonstrated that Dp can induce cell cycle arrest in the G2/M phase through decreasing cyclin B1 and Cdk1 protein expression levels. Additionally, Dp promoted the mitochondrial apoptosis pathway by inhibiting the ERK and NF-κB signaling pathway and activating the JNK signaling pathway [7]. Long [8] studied the impact of anthocyanins extracted from mulberry on the thyroid cancer cell lines SWI1736 and HTh-7. Mulberry anthocyanins significantly reduced the viability of the thyroid cancer cell lines in a time- and dose-dependent manner. Mulberry anthocyanins also enhanced cell apoptosis and autophagy-dependent cell death [8]. Lippert et al. [9] used blueberry anthocyanin extract to treat colorectal cancer in mice. Anthocyanins reduced inflammation and prevented the development of colorectal cancer by reducing the activity of proinflammatory enzymes [9].

Despite the fact that there is much evidence on the health-promoting activities of anthocyanins, a closer look at the methods used in this research reveals that the conclusions about the anthocyanin’s effects are usually based on whole fruit/vegetable extracts containing these phytochemicals rather than their standard solutions. As shown in our previous studies [10,11,12,13,14,15], bioactive phytochemicals in plant extracts are interacting with each other, which impacts their biological activity, so to draw any firm conclusions about the activity of single compounds, they should be used in the form of solutions of purified substances rather than whole-plant extracts.

The objective of this study was to determine the relationships between chemical structure, antioxidant properties and biological effects for structurally different anthocyanidins. The starting point was the determination of antioxidant activities using popular chemical tests (ABTS, DPPH and ORAC). All these tests were performed under physiologically relevant temperature—37 °C. Subsequently, biological activities, such as cytotoxicity, cellular antioxidant activity (CAA), genotoxicity, and ability to protect DNA from oxidative damage, were assessed in HT29 cells. To the best of our knowledge, no such comprehensive redox-related evaluation has been undertaken before for this set of compounds.

## 2. Results and Discussion

### 2.1. Antioxidant Activity Determined by Spectrophotometric Methods (ABTS, DPPH, ORAC)

**Figure 2:**
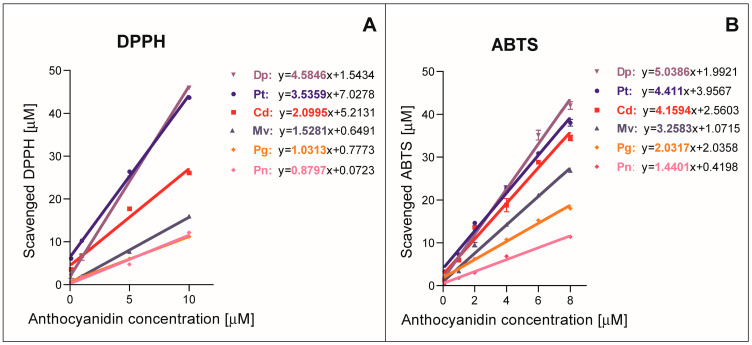
The relationship between concentration of reduced radicals and the concentration of anthocyanidins determined by DPPH (**A**) and ABTS test (**B**) after 10 min of redox reaction at 37 °C. The tangents included in the graph equations represent stoichiometric values n_10_. The results are means ± SD of three independent determinations. Abbreviations used: Dp—delphinidin chloride, Pt—petunidin chloride, Cd—cyanidin chloride, Mv—malvidin chloride, Pg—pelargonidin chloride, Pn—peonidin chloride.

ABTS and DPPH assays have been widely used to determine the antioxidant activity of both plant extracts and pure compound solutions. In most of the published studies, the results of these tests are expressed as EC50 values (i.e., the concentration necessary to scavenge 50% of ABTS•+ or DPPH• radicals) or as TEAC values (trolox equivalent antioxidant capacity). In this study, a stoichiometric (n_10_) value was used [16], which describes the number of oxidant molecules (ABTS•+ or DPPH• radicals) reduced by one molecule of tested antioxidant after 10 min of reaction, and thus it also considers kinetic aspects of these redox reactions. The concentration response lines obtained after 10 min and calculated on their basis stoichiometric values are presented in Figure 2. The higher the stoichiometric value (n_10_), the higher the antioxidant activity. The results indicate the same trend for the two applied assays, where antioxidant activity of tested compounds increased in the following order: Pn < Pg < Mv < Cd < Pt < Dp.

The relationship between determined antioxidant activity and the structure of tested anthocyanidins is evident. The more hydroxyl groups in the B ring, the higher the antioxidant activity of the tested compound (Pg < Cd < Dp). The replacement of a hydroxyl group with a methoxy group decreased the expected antioxidant activity (Mv < Pt < Dp) and (Pn < Cd). It has been determined that the 3′ and 4′ hydroxyl groups in the structure of anthocyanidins are crucial for the antioxidant capacity of these compounds. These groups can be easily oxidized by donating one or two electrons [17,18].

Our results are in agreement with those obtained by De Lima [19], who also compared the antioxidant activity of delphinidin, cyanidin and pelargonidin chlorides using the DPPH test. Despite experimental differences (temperature −20 °C and time of incubation with DPPH radical 5 min), the same order in the antioxidant activity has been determined: Pg < Cd < Dp. It was concluded that the greater the number of hydroxyl groups in the B ring, the greater the antioxidant activity of anthocyanidins [19]. The results of several previous studies also proved that Dp shows the strongest antioxidant activity among anthocyanidins. These properties of Dp are attributed to the presence of three hydroxyl groups in the B ring [17,18]. Pt and Cd also have hydroxyl groups in the 3′ and 4’ positions, which has a positive effect on their antioxidant activity. Mv, Pg and Pn, which have a single hydroxyl group in the B ring, display lower reducing properties [19].

**Figure 3:**
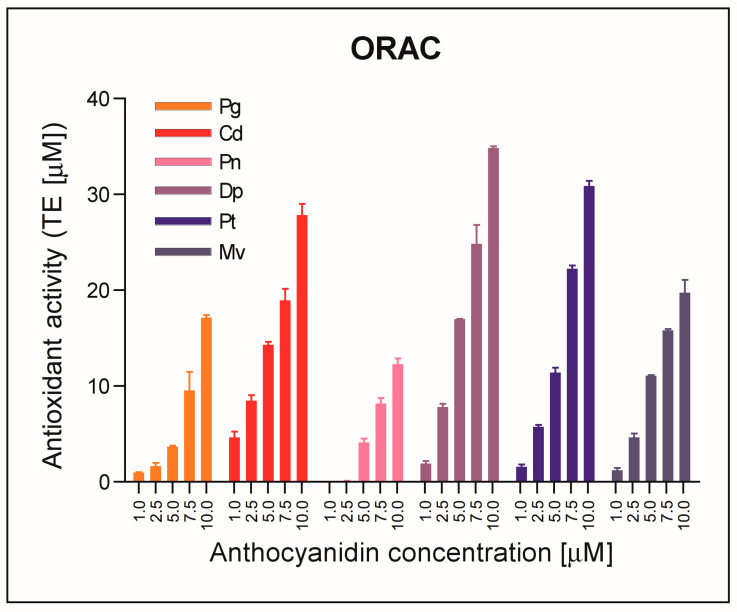
Antioxidant activity of tested anthocyanidins determined using fluorometric ORAC test. The results are means ± SD of three independent determinations. Abbreviations used: Dp—delphinidin chloride, Pt—petunidin chloride, Cd—cyanidin chloride, Mv—malvidin chloride, Pg—pelargonidin chloride, Pn—peonidin chloride.

Another assay employed in our study to compare antioxidant activity of pure anthocyanidins was the ORAC (oxygen radical antioxidant capacity) assay. The results obtained using the ORAC assay confirmed the results obtained by spectrophotometric tests (ABTS and DPPH). According to the assay results, antioxidant activity of tested anthocyanidins increased in the following order: Pn < Pg < Mv < Cd < Pt < Dp (Figure 3).

Wang et al. [20] also tested the antioxidant capacity of anthocyanidins using the ORAC test. They showed that Cd had the highest antioxidant activity among the five tested anthocyanidins. Next came Dp, then Mv, Pn, and Pg. Their results differ from ours, but they might have used different reaction conditions (temperature and incubation time were not specified in the methods section) in the ORAC assay, and they used DMSO (dimethyl sulfoxide) instead of 10% EtOH to prepare standard solutions of anthocyanidins. The relationship between the structure of anthocyanidins and their antioxidant activity was also assessed. Hydroxyl and methoxy substituents have been confirmed to affect the free radical scavenging capacity of anthocyanidins. Anthocyanidins with one hydroxyl group in the 4′ position, including Pg, Mv and Pn, were characterized by the lowest antioxidant activity. Cd, which has two hydroxyl groups in the B ring, exhibited the highest activity among the anthocyanidins tested. However, Dp, which has three hydroxyl groups, had lower antioxidant activity than Cd and the authors did not try to explain the possible cause of this result [20].

### 2.2. Cellular Antioxidant Activity (CAA)

The cellular antioxidant activity (CAA) assay was applied to compare the antioxidant properties of tested anthocyanidins in the cell model. This assay is more biologically relevant than other popular assays used for determination of antioxidant activity because it simulates the redox conditions existing in the human body, including physiological temperature and pH conditions, uptake, transport, and cellular metabolism of antioxidants [21]. The human colon adenocarcinoma HT29 cell line was chosen because it can be used as a model of the alimentary tract, a tissue being in direct contact with ingested food components, including phytochemicals such as anthocyanidins. The assessment of antioxidant effects in the CAA test is based on the ability of antioxidants to inhibit oxidation of the specific probe-2,7-dichlorodihydrofluorescein (DCFH). The results of fluorescence measurements are recalculated into so-called CAA values, as described in the Materials and Methods section.

**Figure 4:**
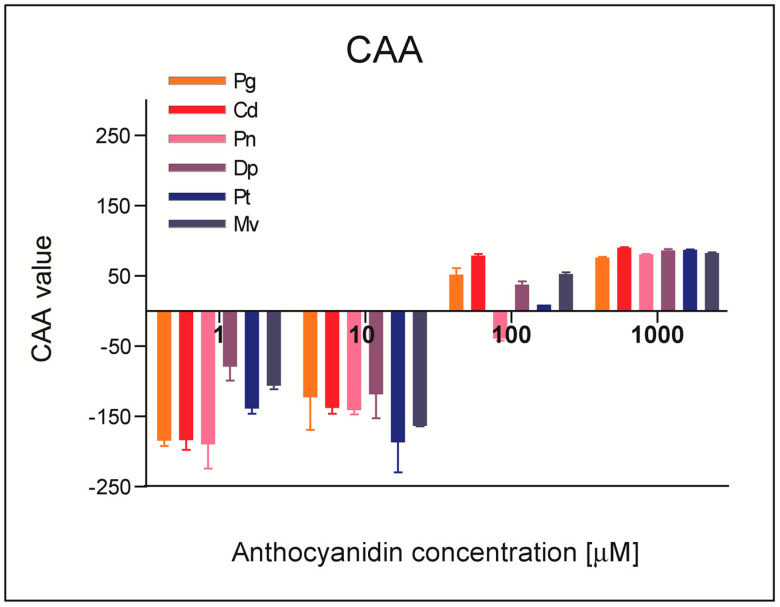
Cellular antioxidant activity of anthocyanidins determined in HT29 cells following 1 h exposure. Results are means ± SD of three independent experiments. Abbreviations used: Dp—delphinidin chloride, Pt—petunidin chloride, Cd—cyanidin chloride, Mv—malvidin chloride, Pg—pelargonidin chloride, Pn—peonidin chloride.

As shown in Figure 4, in the case of treatments with lower concentrations (1 μM, 10 μM) of the investigated compounds, CAA values were negative, which means that anthocyanidins at these concentrations did not show antioxidant properties in tested conditions. At higher concentrations, i.e., 100 μM and 1 mM, all tested compounds increased cellular antioxidant capacity to a similar extent. The lack of antioxidant activity at the lower concentrations most probably results from the low bioavailability of hydrophilic anthocyanins. Antioxidant activity of tested anthocyanidins in cells depends not only on their chemical structure but also on their bioavailability and metabolism, which are affected by compound lipophilicity. One of the proven aspects that impacts bioavailability of these compounds is their ability to bind to ABC transporters and as a result disturbing the 3D structure of these transporters. The resulting efflux of anthocyanins from cells diminishes their intracellular concentration, and thus reduces antioxidant properties. Apparently, only high concentrations of studied compounds applied to cells overcome effects of ABC transporters and could affect the redox status of HT29 cells [22].

Chen et al. [23] also studied the cellular antioxidant activity of Dp and Pt by the CAA assay using a rat cardiomyocyte (H9c2) cell line. They showed that the value of Pt CAA units was higher than that of Dp, which indicates a stronger cellular antioxidant activity of Pt for concentrations in the range of 0–10 μM, so the concentrations were used also in our study [23]. The cellular antioxidant activity of Dp and Cd was also tested by Cvorovic et al. [24] using three different human colon cancer cell lines: human colorectal carcinoma (Caco-2), human colorectal adenocarcinoma metastasis (LoVo) and doxorubicin-resistant metastasis human colorectal adenocarcinoma (LoVo/ADR) cells. In Caco-2 cells, both tested compounds showed antioxidant activity, inhibiting the production of free radicals. For the LoVo cell line, only Cd turned out to have antioxidant potential. Dp in the LoVo and LoVo/ADR cell lines acted as a pro-oxidant, as was also observed in our study for low concentrations of anthocyanidins [24].

### 2.3. Inhibition of Cell Growth Determined by MTT Test

Determination of cell growth inhibition using the MTT (thiazolyl blue tetrazolium bromide) test is a standard first step in the assessment of biological potential of tested substances. The human colon adenocarcinoma HT29 cell line in our study played a dual role: firstly, as a model of frequent human cancer to compare the postulated ability of different anthocyanidins to inhibit cancer cell growth; and secondly, as mentioned earlier as a model of the alimentary tract, a tissue being in direct contact with ingested food components, including phytochemicals such as anthocyanidins.

**Figure 5:**
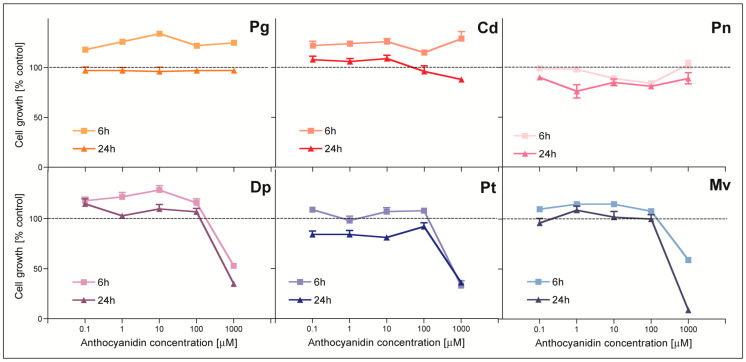
Growth inhibition of HT29 cells determined by MTT test after 6 (squares) and 24 (triangles) h exposure to tested anthocyanidins. Results represent means ± SD of three independent experiments. Abbreviations used: Dp—delphinidin chloride, Pt—petunidin chloride, Cd—cyanidin chloride, Mv—malvidin chloride, Pg—pelargonidin chloride, Pn—peonidin chloride.

The obtained results showed that three of the tested anthocyanidins (Pg, Cd, Dp) in a shorter incubation period (6 h) caused significant cell growth stimulation (Figure 5). The common feature of these compounds is the fact that they do not have methoxy groups in their structure. Among these three compounds, only Dp displayed the ability to inhibit cell growth and only at the highest (non-physiological) concentration (1 mM). Pn, which contains one methoxy group, did not induce the stimulation of cell growth after 6 h incubation; after 24 h treatment, it even caused slight growth inhibition. While in the case of anthocyanidins with three substituents in the molecule (Dp, Pt, Mv), the cytotoxic effect was stronger with each methoxy group added to the molecule (Dp < Pt < Mv), Mv turned out to be the most cytotoxic compound among this group of anthocyanidins. These results showed that there is a strong relationship between the structure and biological activity of the tested anthocyanidins. It is believed that the presence of substituents at the 3′, 4′ and 5′ positions affects the cytotoxicity of anthocyanins. In the MTT test, it was the anthocyanidins with three substituents in the 3′, 4′ and 5′ positions that showed the greatest ability to inhibit cell growth.

Yoshino et al. [25] studied the cytotoxic effect of five anthocyanidins (Dp, Mv, Cd, Pn, and Pg) against the human leukemia cell line HL-60. Ability to inhibit cell growth was measured using the XTT assay, where the cells were treated for 48 h with the tested anthocyanidins. A cytotoxic effect was observed in an ascending order of Pg < Cd < Pn < Mv < Dp. The results of these studies correlate with the results obtained by us in the MTT test. Cvorovic et al. [24] studied the cytotoxic activity of selected anthocyanidins in the Caco-2 colorectal cancer cell line and LoVo colorectal cancer. Dp, Cd, Mv and Pg were used in this research. An MTT test was performed where the cells were treated for 68 h with anthocyanidin solutions. Under these conditions, Dp and Cd were cytotoxic to LoVo cells, while Mv and Pg did not inhibit the growth of any of the cell lines used in the study [18]. Meiers et al. [26] studied the effect of anthocyanidins and their glycosides on the human vulvar carcinoma cell line A431 and the multicellular lung tumor line LXFL529L. Cd, Dp and Mv chlorides as well as cyanidin-3-galactoside and malvidin-3-glucoside were used in the study. The effect of anthocyanins on cell growth was determined by the sulforhodamine B assay where cells were treated for 3 days with the tested compounds. Dp and Cd turned out to be cytotoxic to both cell lines. Dp most effectively inhibited cell growth. Mv only inhibited the growth of A431 cells, but had a weaker effect than Dp and Cd. The glycosides had no cytotoxic effect on any of the cell lines. Data from the sulforhodamine B test suggest that the presence of hydroxyl groups in the B ring is important for cell growth inhibition. Dp, which has three free hydroxyl groups in its structure, showed the highest cytotoxicity. Decrease numbers of hydroxyl groups in the B ring, as exemplified by Cd, reduced cytotoxicity. The presence of sugar residues inhibited the cytotoxic activity of anthocyanins [26].

### 2.4. Genotoxicity and Protection of DNA from Oxidative Damage

It has been shown that antioxidants may protect DNA from H_2_O_2_-mediated damage; however, some may escalate oxidative damage caused by H_2_O_2_, or may even be genotoxic themselves [10,11]. The comet assay, a useful method for detecting DNA strand breaks at the single-cell level, was used by us to study the impact of tested anthocyanidins on DNA integrity.

**Figure 6:**
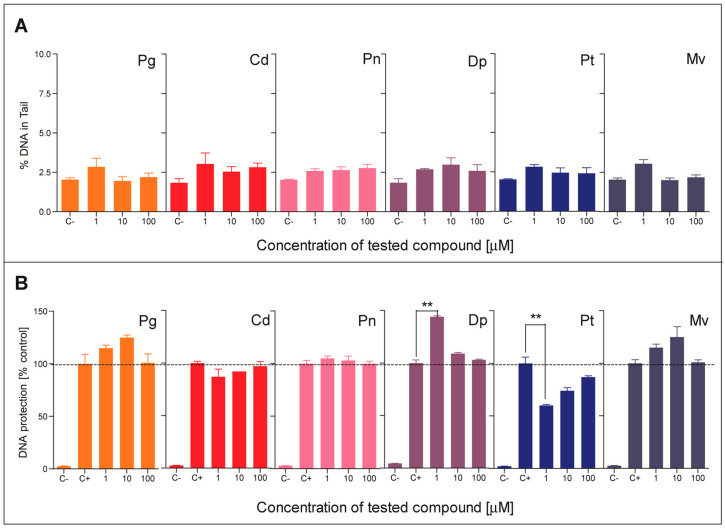
Genotoxicity of tested anthocyanidins expressed as %DNA in comet tail evaluated in HT29 cells (**A**). The ability of tested antioxidants to protect DNA of HT29 cells from H_2_O_2_-induced oxidative damage, expressed as %DNA in comet tail (**B**). Results represent means ± SD of three independent experiments. Negative control (C−)—non-treated cells, positive control (C+)—cells treated with 150 μM H_2_O_2_. Significantly different values determined by one-way ANOVA with Dunnett’s test are marked as (**) *p* < 0.01. Abbreviations used: Dp—delphinidin chloride, Pt—petunidin chloride, Cd—cyanidin chloride, Mv—malvidin chloride, Pg—pelargonidin chloride, Pn—peonidin chloride.

Obtained results (Figure 6A) showed that under the treatment conditions used, none of the investigated anthocyanidins expressed any genotoxic activity towards HT29 cells, regardless of the concentration. The compounds differed, however, when the comet assay was used to determine their ability to protect DNA from oxidative damage caused by H_2_O_2_ (Figure 6B). The significant decrease in DNA fragmentation following 1 h exposure to 150 μM H_2_O_2_ was observed only for cells previously treated for 24 h with Pt at 1 μM concentration. In contrast, Dp at 1 μM significantly potentiated the genotoxic effect caused by H_2_O_2_. These slight genotoxic effects were not reflected by increased cytotoxicity, which suggests that DNA repair systems successfully removed DNA lesions induced by H_2_O_2_ in cells that were pre-exposed to antioxidants.

## 3. Materials and Methods

### 3.1. Chemicals and Reagents

The following anthocyanidins were used in the study: pelargonidin chloride (Pg, purity (HPLC) ≥ 97%); cyanidin chloride (Cd, purity (HPLC) ≥ 96%), peonidin chloride (Pn, purity (HPLC) ≥ 97%), delphinidin chloride (Dp, purity (HPLC) ≥ 97%), petunidin chloride (Pt, purity (HPLC) ≥ 95%) and malvidin chloride (Mv, purity (HPLC) ≥ 97%) from Extrasynthese (Genay Cedex, France). We purchased from Sigma-Aldrich (Taufkirchen, Germany): 1-diphenyl-2-picrylhydrazyl (DPPH, purity ≥ 98% (HPLC)), 2,2-azinobis-(ethyl-2,3-dihydrobenzothiazoline-6-sulfonic acid) diammonium salt (ABTS, purity ≥ 98% (HPLC)), sodium thiosulfate, thiazolyl blue tetrazolium bromide (MTT, purity ≥ 97.5% (HPLC)), hydrogen peroxide (H_2_O_2,_ concentration 35%), phosphate-buffered saline (PBS, quality level 200) and dimethyl sulfoxide (DMSO, purity (HPLC) ≥ 99.7%), McCoys’ 5A medium (modified, with sodium bicarbonate, without L-glutamine, liquid, sterile-filtered, suitable for cell culture), fetal bovine serum (FBS, non-USA origin, sterile-filtered, suitable for cell culture), penicillin–streptomycin (solution stabilized, with 10,000 units penicillin and 10 mg streptomycin/mL, 0.1 μm filtered, BioReagent, suitable for cell culture), low-melting-point agarose (LMP agarose, molecular grade), sodium chloride (NaCl, purity ≥ 99.0%), sodium hydroxide (NaOH, purity ≥ 97.0%), ethylenediaminetetraacetic acid (EDTA, purity 99.4%), 2-amino-2-(hydroxymethyl)-1,3-propanediol (Trizma-Base, purity ≥ 99.8%), Sybr Green I nucleic acid gel stain (concentration 10,000 × in DMSO, and Triton X-100 (laboratory grade). An ORAC assay kit was obtained from Abcam (UK). HPLC-grade methanol was from Merck (Darmstadt, Germany). Normal-melting-point agarose (NMP agarose, molecular grade) was from Bioline (Memphis, TN, USA). The OxiSelectTM Cellular Antioxidant Assay Kit was from Cell Biolabs, Inc. (San Diego, CA, USA). Water was purified with a QPLUS185 system from Millipore (Burlington, MA, USA).

### 3.2. Cell Culture

HT29 (human colon adenocarcinoma) cells purchased from the ATCC were grown in McCoy’s medium supplemented with L-glutamine (2 mol/L), sodium pyruvate (200 g/L), fetal bovine serum (100 mL/L), and antibiotics (100 U/mL penicillin and 100 g/L streptomycin). Cells were maintained at 37 °C under a humidified atmosphere with 5% CO_2_ in a Smart cell incubator (Heal Force, Shanghai, China) as described before [27]. Cultured cells were regularly checked for mycoplasma contamination using a Universal Mycoplasma Detection Kit from ATCC (Manassas, VA, USA).

### 3.3. Antioxidant Activity by Spectrophotometric Methods

The colorimetric determination of antioxidant activity was carried out by standard SET mechanism assays employing ABTS and DPPH radicals as described previously [16,28] with minor modifications. Briefly, stock solutions of radicals were diluted in methanol before measurements until absorbance amounted to 1.00 ± 0.02 at λ = 734 nm in the case of the ABTS radical and 1.00 ± 0.02 at 515 nm for the DPPH radical. All reactions were carried out in 48-well plates at 37 °C. Stock solutions of anthocyanidins were prepared in analytical grade ethanol and then diluted with deionized water to a concentration of 10 mM. Then, stock solutions of antioxidants were diluted to concentrations falling within a linear range of the assay. The ABTS solution (1 mL) was mixed with solutions of anthocyanidins (30 μL). The absorbance of the mixtures was measured at 734 nm after 10 min of incubation in 37 °C. The DPPH solution (1 mL) was mixed with solutions of anthocyanidins (30 μL) and the absorbance was measured at 515 nm after 10 min. All absorbance measurements were performed with a Tecan Infinite M200 spectrophotometer (Tecan Group Ltd., Männedorf, Switzerland). The results of antioxidant activity determinations for spectrophotometric tests were expressed as stoichiometry values (n_10_), as described by Kusznierewicz et al. [15]. This parameter was determined as a regression coefficient, which was defined as the tangent of the line describing the relationship between concentrations of a radical scavenger and concentrations of the tested antioxidant present in the mixture after 10 min of reaction (n_10_). The concentration of radicals scavenged by the tested antioxidants in reaction media was calculated with the use of the Beer–Lambert–Bouguer law (Beer’s law).

The ORAC (oxygen radical absorbance capacity) test is a fluorescent method using the HAT mechanism. In this method, a fluorescent probe is oxidized in the reaction with reactive oxygen species (ROS) to a form that does not emit fluorescence. Antioxidants present in the sample are able to inhibit the decrease in probe fluorescence. [20]. For the determination of antioxidant activity using the ORAC assay, first, 150 μL of fluorescein probe (0.4 nM in PBS) was added to inner wells of a 96-well black fluorescence plate. The outer wells were filled with 150 μL of deionized water and were not used in the measurement. Next, 25 μL of tested samples or standard solutions of trolox were added to each well and the plate was incubated for 30 min in 37 °C in a plate reader. After the incubation period, 25 μL of AAPH solution (153 mM) was added to each well in order to start the oxidation of the fluorescent probe. The fluorescence was measured every minute for 60 min at λ_ex_ = 485 nm and λ_em_ = 528 nm using the Tecan Infinite M200 spectrophotometer (Tecan Group Ltd., Switzerland). The plate reader generated the plot of fluorescence change in time for each sample. To determine the antioxidant capacity of the tested compounds, the area under the curve (AUC) for the obtained plots was calculated for tested samples and for standard solutions of trolox. To calculate the antioxidant activity of the tested samples, standard curves for trolox were plotted and antioxidant activity of the tested anthocyanidins was expressed as trolox equivalents (uM).

### 3.4. MTT Cytotoxicity Test

The MTT test was performed to assess the ability of tested anthocyanidins to inhibit the growth of HT29 cells, as described before [28]. Briefly, HT29 cells were seeded in 96-well tissue culture plates (10^4^ cells per well in 0.15 mL of medium). The cells were allowed to settle for 24 h at 37 °C. Then different concentrations of tested anthocyanidins were added to the cell culture medium and incubated for 6 or 24 h. Final concentrations of compounds in culture medium ranged from 100 nM to 1 mM. In the cases of shorter exposure, the medium was aspirated from the wells, replaced with 0.2 mL of fresh medium, and the cells were incubated at 37 °C until 24 h of the total incubation time. After 24 h of incubation, MTT solution (4 g/L) was added (0.05 mL per well) and the multiwell plate was incubated for another 4 h at 37 °C. Finally, medium was carefully removed from wells and formazan crystals formed by metabolically active cells were dissolved in 0.05 mL of DMSO. The absorbance of resultant solutions was measured at 540 nm with the Tecan Infinite M200 plate reader (Tecan Group Ltd., Switzerland). Three independent replicates of each treatment were performed. Cytotoxicity was expressed as percentage growth inhibition of cells exposed to tested antioxidants compared to control cells treated with the appropriate volume of solvent only, whose growth was regarded as 100%.

### 3.5. CAA Assay

The cellular antioxidant activity (CAA) of investigated compounds in HT29 cells was studied by CAA assay using the OxiSelectTM Cellular Antioxidant Assay Kit (Cell Biolabs, Inc., USA) according to the producer’s manual, as described before [10]. Briefly, HT29 cells were seeded in black 96-well tissue culture plates with transparent bottoms dedicated to fluorescence measurements (3 × 10^4^ cells per well in 0.2 mL of medium). The cells were allowed to settle for 24 h at 37 °C and then were treated with 0.05 mL of different concentrations of anthocyanidins for 1 h. Final concentrations of investigated compounds ranged from 1 to 1000 μM. Control cells were treated with the corresponding solvent. Emission of fluorescence at 538 nm was measured every 5 min for 1 h after excitation at 485 nm using the Tecan Infinite M200 spectrophotometer (Tecan Group Ltd., Switzerland). Three independent replicates of each treatment were performed.

### 3.6. Genotoxic Effects and the Ability to Protect DNA from Oxidative Stress Determined by Comet Assay

Genotoxic properties of tested anthocyanidins and their ability to protect DNA from oxidative stress were determined using a comet assay according to the procedure described before [10,11]. Briefly, in the first case, HT29 cells were seeded in 24-well tissue culture plates (10^5^ cells per well in 1.8 mL of McCoy’s medium) and left to settle for 24 h at 37 °C. The next step involved the treatment of cell cultures with different concentrations (from 1 to 100 μM) of tested anthocyanidins for 24 h. In the case of negative controls, the cells were treated with the solvent only, while positive control cells were treated with 150 μM H_2_O_2_ solution for 1 h. For the experiments in which the protective effect against oxidative stress was assessed, an additional step was to treat the cells with 150 μM H_2_O_2_ solution for 1 h. After treatment, the cells were detached using 0.2 mL of trypsin (0.5 g/L) solution. The cell suspensions were centrifuged (100× *g*, 5 min, 4 °C). The cell pellets were washed with 1 mL of PBS and centrifuged (100× *g*, 5 min, 4 °C) again. After centrifugation, approximately 40 μL of the supernatant was left in the tube to resuspend cells. The cell suspension (30 μL) was mixed with 150 μL of 0.5% LMP (low melting point) agarose. Finally, 30 μL of this mixture was placed as two spots on a microscope slide precoated with 1% normal-melting-point agarose (NMP agarose) and left to set on an ice-cold tray. Three slides with two repetitions on each were prepared for every concentration of the tested anthocyanidins. After overnight lysis in a high-salt alkaline buffer (pH 10), the slides were left in electrophoresis buffer for 20 min before electrophoresis. Electrophoresis was conducted at 26 V and 300 mA for 30 min in darkness at 4–8 °C. After electrophoresis, the slides were transferred to neutralizing buffer (for 5 min). This step was repeated twice. Then, slides were washed using distilled water and fixed in 70% ethanol. Next, the DNA was stained with SybrGreen in TE buffer for 20 min. After staining, the slides were washed with distilled water for 5 min. Finally, DNA “comets” were examined under a fluorescence microscope (Zeiss ImagerZ2, Altlussheim, Germany) connected to a computerized slide scanning system (Metafer4, Altlussheim, Germany). Comet analysis involved counting 200 consecutive nuclei per sample. The mean percentage DNA in the comet tail was a measure of genotoxic potency of compounds tested. As for the protection from oxidative damage experiments, results were recalculated and expressed as percentage control, where control cells were treated only with H_2_O_2_.

### 3.7. Statistical Analysis

All values are expressed as means ± SD of three independent determinations. The statistical significance of determinations was examined by one-way ANOVA with Tukey–Kramer test or one-way ANOVA with Dunnett’s test. All statistical analyses were performed using Prism 4.0 software package (GraphPad Software, Inc., Boston, MA, USA). The level of statistical significance was set at *p* ≤ 0.05.

## 4. Conclusions

This study aimed to determine the relationship between structure and biological activity for the six most common anthocyanidins. The tested compounds included delphinidin chloride, petunidin chloride, malvidin chloride, peonidin chloride, cyanidin chloride and pelargonidin chloride. Antioxidant activity tests results obtained by three different methods (ABTS, DPPH, ORAC) showed the same trend, where antioxidant activity increased in the following order: Pn < Pg < Mv < Cd < Pt < Dp. The relationship between the structure of tested anthocyanidins and their activity could be observed. The presence of hydroxyl groups increased antioxidant activity, while methoxy groups had an opposite effect. In the CAA test conducted on HT29 cells, the antioxidant activity was at the same level for the concentration of 1000 μM. For concentrations below 10 μM, anthocyanins showed a pro-oxidative character. Delphinidin, malvidin and petunidin at 1000 μM concentration showed the greatest ability to inhibit the growth of HT29 cells in the MTT test. These anthocyanins have substituents in the 3′, 4′, and 5′ positions, which makes them the most cytotoxic for cells after entering them. Results obtained by comet assay showed that tested anthocyanidins did not have genotoxic potential, and only Pt showed significant ability to protect DNA from oxidative damage in tested conditions. The chemical structure did not show significant influence on this activity. This study proved that the presence of hydroxyl and methoxy groups has a significant influence on biological activities of tested anthocyanidins. The hypothesis that this group of compounds should not be treated as they all have the same biological potential has been confirmed. This knowledge can be used by producers of functional food and dietary supplements while designing products containing anthocyanins. Future research should confirm these findings in more in vitro and then in vivo models to verify whether different biological activities of anthocyanidins can also be observed in different models.

## Data Availability

Not applicable.
